# The Body of Chagas Disease Vectors

**DOI:** 10.3390/pathogens14010098

**Published:** 2025-01-20

**Authors:** Jean-Pierre Dujardin

**Affiliations:** UMR INTERTRYP University Montpellier, IRD, CIRAD, F-34398 Montpellier, France; dujjepi@gmail.com

**Keywords:** Chagas, Triatominae, morphometrics

## Abstract

Morphometry is an effort to describe or measure the morphology of the body, or parts of it. It also provides quantitative data on the interactions of living organisms with their environment, external or internal. As a discipline, morphometrics has undergone significant developments in the last decade, making its implementation more visual and less laborious. Chagas disease vectors, often referred to by the common name of “kissing bugs”, belong to the subfamily Triatominae. Due to their apparent morphological plasticity, they have been the subject of numerous morphometric studies. Most of these have been applied taking into account the particularities of this group of vectors, such as domesticity (synanthropy), food preferences, dispersal ability, insecticide resistance, as well as some taxonomic issues. This brief review over nearly three decades is organized here according to the body organs considered by the authors.

## 1. Introduction

In the decades following the discovery of American trypanosomiasis by Carlos Chagas [1], control efforts quickly focused on vector control. On the one hand, there was no known problem of insecticide resistance [2], and on the other hand, infection occurred mainly within human habitats where vectors also bred. Two main strategies were considered, habitat improvement and the application of insecticides in human structures. Habitat improvement, although never convincing as a large-scale solution, is generally a highly desirable measure. However, the use of insecticides over very large areas in a few years has made it possible to sustainably reduce vector transmission. This historic success was possible thanks to major international programs developed successively in the Southern Cone of Latin America, in the Andean region, and in Central America. Thus, some of the most important vectors, *Triatoma infestans* in the Southern Cone and *Rhodnius prolixus* in the Andean region and in Central America, have been dislodged from human structures, leaving today only a few wild foci under surveillance. However, epidemiological surveillance must be maintained not only for them, but also for other species of Triatominae [3]. Indeed, some other species adapt to colonize human spaces, perhaps a collateral damage induced by the elimination of the most synanthropic vectors or a previously undetected natural behavior of some Triatominae [4].

In its past and recent development, the fight against Chagas disease vectors has repeatedly encountered the same two particular problems. One is the level of adaptation of the insect to the human habitat, as well as to that of domestic animals. In the literature on Chagas disease vectors, we almost inevitably find terms such as “domiciled” or “domestic”, “peridomestic”, “sylvatic”, all qualifiers that reflect ecological diversity [5]. The other problem is related to both the ecological and taxonomic diversity of vectors [6,7], a situation that can be recognized in the use of terms such as “main vectors”, “secondary vectors”, “potential vectors”, etc. All species of Triatominae are possible vectors simply because they are hematophagous, but, depending on the level of adaptation of a vector to human habitat, not all of them have the same epidemiological importance. The ability of insects to colonize homes varies according to species, but it can also vary within the same species according to geography [8].

Thus, in many situations, the species identity of the vector and its degree of adaptation to humans are crucial elements to know in the development of control strategies. Many techniques can help to resolve these two questions, but all are relatively costly in terms of men and machines, hindering their use as a routine activity in entomology laboratories. Early morphometric studies applied to these problems showed that helpful answers could be provided quickly and inexpensively by morphometric analyses [9], based on techniques that were easily adopted by any laboratory with a computer and optical devices. These studies were themselves stimulated by an important evolution in the discipline of the morphometric itself [10].

The traditional method of collecting body measurement data is to estimate the linear distance between anatomical landmarks. In entomology textbooks, this method has frequently been applied to a few specimens without giving much indication of intraspecific variability [11]. The multivariate morphometric approach [12] was applied to Triatominae in the 1990s [13,14,15,16]. This approach requires larger samples of biological material because it takes into account variation (variance) in measurements. With an acceptable estimate of variances, this approach can produce a synthetic representation of many measurements, thus creating a powerful tool for distinguishing closely related species or groups [17].

In addition to improving discrimination between taxa, the multivariate techniques were also used to tentatively separate the two main properties of the morphometric data: size and shape [18]. However, the different methods to separate these two metric properties could produce different results, and they did not allow to visualize shape differences. These two obstacles could be overcome thanks to the use of anatomical landmark coordinates instead of linear distances between them [10].

Two different statistical approaches use the relative position of coordinates of points: (i) the outline (or pseudo-landmark)- [19] and (ii) landmark (and semi-landmark)-based [20,21] analyses. The former approach, slightly anterior to the latter, is restricted to the situations where landmarks are lacking, or too few. Both techniques belong to the geometric morphometrics (or modern morphometrics) as opposed to linear measurements (or traditional) morphometrics [22], and the most frequently used one currently is the landmark-based method. Application of these modern techniques to different species in many groups of insects, and in other organisms as well, has shown that geometric shape is surprisingly powerful in distinguishing species [23,24,25], even cryptic [26], calling into question the concept of sibling species [25,27,28].

Moreover, new routes of investigation have been opened regarding the interaction between the environment and the body shape [23], bringing a new tool for the study of phenotypic evolution [28]. Finally, two other virtues of the morphometric approach deserve to be mentioned: (i) its non-traumatic access to the data (image) allowing the use of other techniques on the same sample, including molecular techniques, and (ii) the possibility of exchanging images via the Internet, allowing one to compare own material with that of others (but see Section 4.1).

Initially, and until today, the main application of morphometrics to Chagas disease vectors has been oriented towards both taxonomic and epidemiological questions. But over time, many studies have contributed to the more general question of the evolutionary importance of phenotypic plasticity. In this brief review, I wish not only to highlight the breadth of information provided by modern morphometry applied to the Chagas disease vectors, but also to show, in broad terms, how it achieved this.

## 2. Methods

All geometric morphometric studies applied to Chagas vectors have used 2D data collected on images of 3D structures. Therefore, a small parallax error is possible. It is certainly not important for the wings of the insect, because the wings are almost 2D structures. However, the heads of Triatominae are closer to 3D structures. A true 3D capture of some body parts is technically possible but requires expensive equipment, which limits applicability to privileged laboratories. This is not the philosophy of morphometry as a tool to support the monitoring of vector control programs, usually under the supervision of many field laboratories with fewer resources.

### 2.1. Morphometric Techniques

#### 2.1.1. Landmarks

Most of the geometric studies applied to Triatominae use the landmark-based approach [20,29]. In common practice, size and shape are derived from a configuration of landmarks collected on a non-articulated part, often a single organ (see, however, [30]). The relative positions of landmarks contain not only information on size, such as distances between landmarks, but also on shape. Of course, a few landmarks on a wing (or any measurable part of the body) do not completely describe the wing, nor the entire body. But if the landmarks are homologous between individuals, a partial capture of shape is sufficient to allow valid comparisons [28]. Anatomical landmarks are homologous in the sense that they are relocatable points, and according to this criterion, various levels of quality have been recognized (see type I, II and III landmarks, having decreasing levels of precision [21]).

#### 2.1.2. Curves

The use of pseudolandmarks, i.e., points describing closed contours (outline-based approach) [19,31], and the use of semilandmarks, i.e., points describing an open curve between two landmarks [32], are less frequent.

The outline-based approach (pseudolandmarks) is best suited to the situations were no or too few landmarks are available, as, for instance, the eggs [33,34,35], but it has been adapted also to fit adult bodies [36] or internal wing cells [37]. Historically, the outline-based method is anterior to the landmark-based approach. It also uses the relative positions of coordinates, the ones collected on the contour of the organ (pseudolandmarks), but the statistical analyses are quite different. Since shape is a function of the relative positions of morphological landmarks, the outline-based method is also part of geometric morphometrics [22].

In the case of non-closed contours (open curves), the semilandmark-based method applies [38,39] and, despite the loss of degrees of freedom, receives the same statistical treatment as the landmark-based method [40].

When the idea is to capture a maximum of shape information, the use of pseudo-landmarks and/or of semi-landmarks may be combined with landmark-based analysis, as, for instance, in a study on New World versus Old World Triatominae [37].

#### 2.1.3. Softwares

With time, many free software became available, like the TPS series of J. Rohlf [41], the Morpheus from D. Slice (https://morphlab.sc.fsu.edu/ accessed on 16 December 2024), some R packages (geomorph [42], morpho [43]), the MorphoJ software of C. P. Klingenberg [44], or our own online software XYOM [45]. For the outline-based approach of geometric morphometrics. I consider the R momocs package [46], SHAPE [47], or again XYOM [45]. For a comprehensive list of currently available software, please consult https://www.sbmorphometrics.org/ (accessed on 16 December 2024).

### 2.2. Morphometric Data

Geometric morphometric data allow a more informative separation, analysis, and visualization of size and shape.

#### 2.2.1. Size

In the landmark-based method, there is a single size estimator, the centroid size (CS). For each configuration of landmarks, the computation of the CS is using the centroid landmark (average X, average Y), using the (squared) distances from this point to each user annotated landmarks [29]. This estimation of size is affected by changes occurring in different orientations, and it is restricted to the configuration of landmarks, thus, of course, to the organ under study. The change in size of an organ is positively correlated to the change in size of the insect’s body, but the size of an organ does not necessarily reflect the size of the insect.

In the outline-based approach, the size used to normalize the coefficients of Fourier is an abstract “semi-major axis of the first ellipse” (SMA). It should be used for size comparisons between individuals or groups, but one generally prefers to use the perimeter of the contour which is generally highly correlated to the the SMA, and much more intuitively understandable. This correlation might decrease, however, in a case of a rough, very sinuous contour.

Geometric data produce richer information, but this does not exclude the relevance of linear measurements in certain situations. However, if we want to compare shapes between individuals or groups using such measurements, it is not recommended to use ratios. It is necessary to employ specific multivariate analyses [18].

#### 2.2.2. Shape

In geometric morphometrics, the concept of shape derives from the relative positions of anatomical landmarks. Various works argue that shape variables are polygenic traits [48,49,50], and that, therefore, their variation under natural conditions could well be explained by genetic drift [24,25,28]. Of course, this does not exclude local environmental factors [51].

The two kinds of shape, landmarks or curves (see above Section 2.1), might reflect also different functional aspects of “shape” (see Section 3.3.2), a question not specifically considered up to now, except for their taxonomic power. In this respect, the two techniques seem to contain an approximately equivalent taxonomic signal [36,37,52], but their statistical treatments are very different. Also very different are the numbers of shape variables produced by these different approaches, as well as the estimation of size. To avoid the “curse of multidimensionality” [53], one technique is to reduce the number of variables by using a representative set of their few first principal components.

#### 2.2.3. Size and Shape

Geometric shape variables are exempt from isometric size change but not from allometric size change. Allometric changes have a direct effect on body proportions (shape). They are produced by different rates of development in different organs or their constituents. Three different levels of allometry are distinguished: static allometry (between individuals of the same age), ontogenetic allometry (due to growth processes), and evolutionary allometry (between taxa) [18].

Thus, part of the geometric shape is a passive change due to variation in size: this part may be called the allometric residue. The effect of this allometric residue could be neglected for interspecific comparisons, where size changes are probably part of the evolutionary divergence between species. But it is important to take it into account, or at least measure it, for conspecific populations [23,28]. Between populations of the same species, size variation is usually due to contingent factors, masking microevolutionary changes reliably detected by shape changes, or interfering with the detection of a possible speciation event [54,55,56].

Generally, it is relevant to evaluate its importance. The simplest procedure for this is to calculate the linear correlation between size variation and shape variation, using as shape variation the first principal component of the shape variables. To include the totality of shape variables in this estimation, one needs to use a multivariate regression on size. Since it is a parametric test, statistical significance is better evaluated by a non-parametric algorithm. The procedure first calculates the observed values of the vector of regression coefficients (one coefficient for each component of shape), multiplying this vector by its transpose to produce a scalar value (which is the sum of squared coefficients). It then permutes the column of sizes a given number of times; at each permutation, it calculates again the regression coefficients (pseudo-coefficients) and the vector of pseudo-coefficients is multiplied by its transpose. After a given number of permutations, it counts the number of times that the pseudo-values were greater than or equal to the observed one, and it divides this number by the number of permutations. This is the risk of error saying that allometry is significant [57]. Other methods use multivariate analysis of covariance (MANCOVA) of shape variables, considering the compared groups as categorical predictors and centroid size as a covariate [58,59]. Such procedure appears to have been used only once in Triatominae [60].

### 2.3. Classification Methods

Classification of methods into distinct units (species, ecotypes) is mainly based on shape variables (instead of size), using both unsupervised and supervised analyses. Because the unsupervised methods make no assumption about the pre-established groups of individuals and because they do not distort the Procrustes distances (shape divergence), they are highly recommendable. Among them, the reference one, i.e., principal component analysis (PCA), has been used in all published works on geometric morphometrics applied to Triatominae. Other methods were less frequently or seldom used, such as Hierarchical clustering and Kmeans clustering, respectively [55,61].

The supervised methods try to reproduce, at best, the classification proposed by the user. Among these methods, Linear Discriminant Analysis (LDA) is the most frequently used one. For the morphometrician, the use of LDA suffers from one important criticism: shape variables are modified during the classification process (divided by the groups variances) so that the Mahalanobis distances may depart strongly from the Procrustes distances. To avoid such shape distortion during the classification process, between-group-PCA has been designed [62], later discredited because of unexpected issues when the number of variables was too large relative to the number of groups to classify [63]. LDA is, however, highly powerful, providing relevant classification, but it remains very sensitive to strongly unequal sample sizes [64] and prone to overfitting when the number of input variables is large relative to the smallest group. Not all studies on Triatominae clearly mention the number of variables used as input to LDA.

Both PCA and LDA could be criticized because they are parametric methods, thus depending on various assumptions related to multinormality and variance structure of the data. However, they are robust and generally provide relevant results. In one study, the maximum likelihood method, although a parametric method, has been suggested, performed on the principal components of shape variables [65]. It has the advantage of being applied to univariate (size) or multivariate (shape) data, but it relies heavily on the unrealistic statistical assumptions of normality. Therefore, the statistical significance of the tests should rely on non-parametric techniques (permutations, bootstraps).

To the exception of one study based on the contour of the adult body [36], non-parametric machine learning methods like K-Nearest Neighbors (KNNs), Artificial Neural Network (ANN), Support Vector Machine (SVM), or Random Forest (RF), all techniques that do not affect the Procrustes distances, have not been used in morphometric studies applied to Triatominae [28].

Machine learning attempts to implement an automated identification system [66,67] are not included in this review because not all descriptors are morphometric data, and they are processed directly by neural networks without separately extracting size and shape.

### 2.4. Reclassification Methods

The usual technique for reclassifying groups proceeds on an individual-by-individual basis: each is reclassified after being removed from the classification model (discriminant analysis, or other). Thus, to reclassify a sample using discriminant analysis (LDA), there are as many LDAs as there are individuals in the sample. And ideally, between each run, the shape variables should be recalculated [68]. The result is a percentage of correct assignments for each pre-established category in the sample.

The question is whether it is possible to account for a correct prediction due to mere chance. Such a special statistic has existed for a long time [69], called total adjusted precision (*TAU*). This *TAU* statistic measures the proportion of correctly assigned individuals in the sample remaining after eliminating (the number of) those correctly assigned by chance. It is valid for any group or species assignment and is calculated as follows:

(1) $$TAU=Nc-\left(\sum_{i=1}^{G}\left(Ni*Pi\right)\right)/\left(N-\sum_{i=1}^{G}\left(Ni*Pi\right)\right)$$

where *N* is total sample size; *Nc* is the total number of cases correctly assigned by the classification method; *Pi* is the prior probability of group membership in the *i*th of the *G* groups; and *Ni* is the number of cases in the *i*th group.

### 2.5. Special Morphometric Comparisons

Size and shape data extracted from linear measurements or coordinate data are generally used to compare distinct taxa. They are also used between different intraspecific entities, such as ecotopes, chromatic variants, geographic areas. In addition, these same techniques are applied to comparisons between sexes or even between different parts of the same individual.

#### 2.5.1. Between Sexes

Sexual size and shape differences are generally studied within species. Sexual size dimorphism was first observed between different ecotopes in *T. infestans*, as well as in *R. domesticus* when moving from field to laboratory conditions [70]. These observations suggested that sexual dimorphism could be a sensitive marker of the environment [71]. Its cause was attributed to an unequal rate of size change between habitats: more pronounced in female specimens, it led to altered sexual dimorphism. Later publications confirmed this observation and included shape in the study of sexual dimorphism (see Section 4.5.1). There are no particular statistics to apply here. Another sexual dimorphism was disclosed and discussed related to the sensory system of the antennae (see Section 3.4), also showing typical changes in the transition from sylvatic to (peri)domestic populations (see Section 4.5.1).

#### 2.5.2. Between Body Sides

To compare the two sides of an insect, i.e., to estimate its symmetry, specialized methods are needed because it is imperative to statistically eliminate measurement error. In fact, quantitative estimation of body symmetry is an important area of morphometrics, and a particular asset: it is a full-fledged application, unmatched by methods using genetic markers. It quantitatively explores the interaction of an organism with its environment, as well as, indirectly, with its internal genetic makeup.

The examination of bilateral symmetry (“matching symmetry”, [72,73]) requires bilateral structures, such as wings, antennae, and legs. The statistical procedure can be adapted for an organ which is symmetric in itself (“object symmetry”, [73]) like the head, the pronotum, or female genitalia of the Triatominae. For such organs, it is also possible to use the same statistical method as for bilateral symmetry after removing landmarks that are common to both sides [61]. Estimating the bilateral symmetry follows the same statistical procedure for size as for shape (the venation architecture of the wing and/or its contour), adapting, however, the number of degrees of freedom [72,74]: for a comprehensive review of the topic, see [73].

Ideally, the estimation of the symmetry of the body or of an organ requires to minimize annotation errors, so that it should be estimated using, preferably, landmarks of type I (wings, female genitalia) (see Section 2.1.1).

The kind of asymmetry which is explored is fluctuating asymmetry (FA), i.e., the variance of bilateral differences, assumed to exist mainly due to environmental stressors (developmental instability) [75,76]. The source of developmental stress can also be genetic (intense selection processes, inbreeding/exogamy, mutations). For instance, more FA could be demonstrated in insecticide-resistant *T. infestans* versus -susceptible ones [61].

The main hypothesis is that FA increases in cases of stress affecting the development of the insect, whatever the cause of the stress. Under this expression, the most frequent stressing factors are environmental ones: higher population density (more competition for food) [71,77], sublethal doses of insecticides [78], nutrition [79], extreme climatic conditions, flight muscle dimorphism [80] or activity [81], parasitism [82], etc.

For the Chagas disease vectors that have been submitted to symmetry analyses, many of the factors mentioned above are the ones distinguishing sylvatic from domestic populations [71], melanic from non-melanic forms of *T. infestans* [55], good flyers from others [80], insecticide-resistant specimens from susceptible ones [61], and survivors from others [78]. Thus, although not *a priori* a heritable character, the symmetry of an insect is a valuable source of information on its adaptation to the local environment. In the case of vectors that are vectors because of their adaptation to human habitation, this type of information, although not always easy to interpret, can be of great help in understanding their level of domestication and improving vector control strategies (see Section 4.5.1).

#### 2.5.3. Between Body Parts

Less frequently, morphometric data are used to compute the correlation between different body parts: linear correlation for the univariate size estimator [83], two-block partial least-squares method (PLS) [84], or the Escoufier coefficient [85] for the multivariate shape descriptor [35,83,86]. The PLS statistics was applied to estimate the covariation of head and wing shapes as a species specific trait [35]. PLS statistics are also applied to estimate the covariation of organ shape with the size of another organ (wing shape and head size, wing shape and pronotum width, see [86]), not to be confused with the estimation of the allometric residue embedded in shape variables (see Section 2.2.3). The same statistical techniques can be used to correlate shape variation with external parameters such as climate variables [51,86,87].

## 3. Body

Different stages (eggs, nymphs, adults) and different body organs (heads, wings, pronotum, legs, genitalia) were subjected to 2D geometric morphometric approach.

### 3.1. Eggs

Traditionally, Triatominae eggs are studied based on linear measurements [88,89,90], using light microscopy and scanning electron microscopy [91]. The geometric approach to egg morphometry uses light microscopy [33,34,35,65]. The egg outline can be treated as a closed contour, including or excluding the operculum. The contour-based approach can be used provided that the egg position can be correctly repeated from one individual to another [31]. The operculum could also be treated separately using semi-landmarks describing an open curve between two landmarks [32,40]. Ecuadorian populations of closely related species, *Panstrongylus chinai* and *P. howardi* are clearly distinguishable by egg outlines, but not convincingly by head shape or wing venation [35]. Even slightly deformed nonviable eggs retain significant taxonomic signals [34].

### 3.2. Nymphs

Because of their epidemiological importance [92] and their more elusive diagnostic characters, nymphs have attracted the interest of morphometricians. Furthermore, because of the incomplete metamorphosis typical of Triatominae, ontogenetic growth could be examined and used as an element of interspecific comparisons.

The projection in a common morphospace of the head shape of successive nymphal stages and of adults allowed to compare the ontogenetic trajectories of four species among the three most important genera of Triatominae: *T. lecticularia*, *T. infestans*, *R. prolixus* and *P. megistus* [93]. A similar approach visualizing the ontogenetic profile of postembryonic head shape development showed that, for *Belminus herreri*, the greatest change occured between the first and second instars [94], while for *T. ryckmani*, largest changes occurred in the first three instars [95].

The head shape of adults and nymphs produced generally concordant results. An intraspecific study (*T. infestans*), however, observed a lower discrimination ability of nymphs between distinct habitats compared to adults [83].

Interspecific comparisons did not observe such a difference between adults and nymphs: the five successive nymphal instars suggested a clear distinction between three of the seven species of the *R. prolixus* complex: *R. marabaensis*, *R. prolixus* and *R. robustus* [96].

### 3.3. Adults

#### 3.3.1. Body Contour

The body outline excluding legs and antennae was examined as a closed contour (outline-based method). This approach produced better reclassification scores than other methods to distinguish the major evolutionary lineages of *T. dimidiata* [36]. However, it depends on image preprocessing (removal of legs and antennae) which could be an obstacle to its diffusion.

#### 3.3.2. Wings

As in most winged insect groups, the wing (forewing for Triatominae) is the organ of choice for morphometric analysis because it can be easily separated from the body, allowing the rest of the body to be used for other analytical techniques. The nearly two-dimensional structure of the wing reduces digitization errors, at least those due to different orientations as is the case for heads (see Section 3.3.3).

Due to the better repeatability of measurements, wing vein geometry is ideal for exploring bilateral symmetry (see Section 2.5.2), where minimizing measurement errors is of crucial importance.

For the same reason, the wings have been used to compare very close, even cryptic or sibling species, an exercise where wing vein geometry has proven powerful [39,52,97,98,99] (see also Section 4.2), as well as to compare intraspecific entities, separated by either space [100] or time [101] (see also Section 4.4.1).

The geometric approach applied to the wing can cover different realities: either the architecture of the internal veins or the outline of the wing or its parts (rigid and membranous parts).

The venation pattern is typically analyzed by the landmark-based approach, all of them landmarks of type I [29], i.e., landmarks having the highest level of relocability from one individual to another.

The external outline of the forewing (or the outline of some internal cells [37]) can be approximated by the pseudolandmark technique (outline-based approach). For the Triatominae, the outline of an internal part (rigid, membranous) contains some landmarks so that it can also be approximated by the use of semi-landmarks [37,39]. The shape described by these techniques does not use or uses very few type I landmarks.

It is, however, not certain that these different forms respond in the same way to external factors, in particular by comparing the external contour of the wing and its internal venation architecture. The external contour of the wing (contour-based shape), as well as the size of the wing itself, can present changes visible to the naked eye [102]. Intuitively, these changes mechanically affect the insect’s flight capacity. On the contrary, it is less obvious to imagine that invisible or subtle changes in the internal venation of the wing have an impact on flight capacity. For any flying insect, the flight function actually depends on various constituent elements of the wing apparatus as a whole (surface and external dimensions of the wing, shape of the wing contour, dimensions of the eye, spacing of the eyes, presence or activity of wing muscles, antennal sensory system, nutritional status, body weight, etc.). Such a complex system, reflected by changes in body parts other than the wings themselves [80,103], probably does not depend much on subtle variations in the architecture of forewing venation.

In other words, the internal venation architecture variation is not likely to induce by itself a different flying capacity, nor is it likely to have any adaptive value: acording to [104], wing venation, as a by product of natural selection, is almost a neutral character.

#### 3.3.3. Head

The above considerations about shape as landmarks and shape as outline may be important for wings, but not for heads. For heads, indeed, landmarks probably tell the same story as those of the outline, because the landmarks of the head are part of its outline (as captured by the image of the head).

Unlike the internal venation of the wings, the shape of the head seems more likely to vary as an adaptive trait. The head of Triatominae is a composite, multifunctional organ, bearing a proboscis (rostrum), two antennae and two eyes, two ocelli, all structures that allow the insect to evaluate its environment and respond to a wide range of stimuli. Head shape variation, in addition to being affected by genetic drift in isolated populations [38,83], could be more directly related to external parameters such as feeding habits [60,83,105,106,107], environmental factors detected by the antennae [83,108], or light exposure affecting eye size [27]. Other associations have been highlighted, such as the ability to fly and the separation of the eyes, or simply the size of the head [102,103].

Assuming that the head is attributed a greater correspondence to environmental factors, this organ then turns out to be the one of choice to explore intraspecific variation related to different food sources (see Section 4.6.1) or habitats [83,109], thus leading to the possible detection of different ecotypes [38,108].

In any case, the shape of the head, derived from linear measurements or a geometric approach, is generally distinct between species (see Section 4.2) and has been used as an additional argument for the description of new species [110]. Another advantage of the head, and not the least, is that it allows to include the nymphs in a morphometric study (see Section 3.2).

However, the use of the head alone [110,111,112,113] is not encouraged without some precautions. For a morphometrician, the head can be a problematic organ for three reasons: (i) the landmarks describing the geometry of the head are of lower quality (there is no type I landmark), (ii) its volume makes it difficult to offer each specimen the same head orientation, generating errors in the annotation of the landmarks, and (iii) the landmarks actually cover the left and right parts of the body, thus including possible asymmetries (object symmetry) in their intergroup variation. Due to its lack of type I landmarks alone, the head is less recommendable than the wings for the study of asymmetry, where geometric capture must be as rigorous as possible (see Section 2.5.2). Authors generally take the precaution of either averaging the bilateral homologous landmarks or testing only one side of the head.

#### 3.3.4. Head and Wing

A combined morphometric analysis of heads (adults and fifth instar nymphs) and wings was used to study the mobility of *T. infestans* between poultry and goat pens: the authors observed a lack of covariation in shape between the two organs [83]. Subsequent morphometric studies including both heads and wings often highlight the difference in information provided by the two organs (but see [39]), either within species [56,107,109] or between them [97,114].

For the *R. prolixus* complex, heads appeared to be more discriminating than wings [99], while for the *T. sordida* subcomplex, especially the *T. sordida* and *T. garciabesi* comparison, wings were more informative [98,114]. In a phylogenetic study focusing on the *T. brasiliensis* species complex (*T. brasiliensis*, *T. juazeirensis*, *T. sherlocki*, *T. melanica*, *T. lenti*, *T. petrocchiae*), adding *T. melanocephala*, *T. vitticeps*, and *T. tibiamaculata* species, the head shape information did not correlate well with the wings, and the shape variation of the wings showed more agreement with genetic variation [97]. Within the highly morphologically variable and ecologically flexible *R. ecuadoriensis* from Ecuador and Peru, the geometric shapes of head and wings suggested different patterns of variation, with the wing shape closer to the genetic classification [109]. Such discrepancies could be related to the taxa examined, or to the properties of the organ itself (head, wing). A recommended precaution in such a comparison between head and wings is to also include the comparison of measurement errors (see Section 4.1).

#### 3.3.5. Pronotum

In Triatominae, linear measurements of the pronotum (length, width) have traditionally been associated with species descriptions [11,115]. Its study with geometric techniques used the using semilandmarks approach due to the scarcity of valid landmarks. Its use is recent, and publications on this organ combined its information with that of the head [38] or wings [39,98]. As for heads, interference of object symmetry in final shape variables can be avoided by averaging the sides or by considering only one side.

Pronotum shape appears to have good discriminatory power in the *T. sordida* subcomplex. However, the topology (NJ trees) of shape-based classifications, similar when derived from heads or wings, was different when based on the pronotum [98]. The pronotum shape was also effective for distinguishing different haplotypes of *T. pallidipennis* [38].

Since a broad thorax may imply highly developed forewing muscles [116], the pronotum has been included in studies related to flight dispersal ability. Between two geographic lineages of *T. garciabesi*, the size of the pronotum, unlike its shape, showed a different trend than that observed for the head and forewings [39].

Visualization of the body contours of the three haplogroups of *T. dimidiata* suggested that the pronotum is the most variable body part between haplogroups [36].

#### 3.3.6. Legs

Along with the head and pronotum, the legs represent another organ that can be examined in nymphs and adults. The idea is that walking is certainly an active dispersal mechanism, especially for nymphs, and thus likely to play an important role in exchanges between domestic and peridomestic sites [92].

There is a pioneering work that paves the way for this morphometric application where the relative dimensions of legs and leg segments explore the locomotion of *T. infestans* [117]. The authors use both linear measurements (femur, tibia) and the geometric approach (meron, coxa, trochanter) of the right side of *T. infestans*, comparing forelimbs, midlimbs, and hindlimbs, and calculating the correlation between them.

In the future, legs could be a candidate for asymmetry studies, in spite of not having landmarks of type I (see Section 2.5.2).

#### 3.3.7. Female Genitalia

Another morphometric study examined, for the first time, the geometry of female genitalia to explore their variation between genera and species [118]. This is an organ limited to one sex of adults, thus unable to include nymphs, unable to explore sexual dimorphism, but it presents an interesting feature: according to the authors, all landmarks were type I [21]. It is then a possible candidate for reliable object symmetry analyses [73].

### 3.4. Antennae

This organ deserves special attention because of its particular reading, based on the density of micro-organs that line its segments. Traditionally, relative linear dimensions of antennal segments are used in species description [11], but, whether by electron or optical microscope, other antennal characteristics are measurable. The four segments of the antennae are covered with specialized sensilla (setae, basiconic, trichoids with thick and thin walls) capturing chemical, physical, or mechanical signals [119]. Their counting (and length) under the optical microscope produces meristic variables (instead of continuous variables) defining the antennal phenotype (AP). The AP was insect developmental stage specific and distinct between genera and species [120,121] (but see [35]), distinct between haplotypes of *T. dimidiata* [122], and distinct between cytotypes (chromosomal variants) [123] or ecotypes [108] of *T. infestans*.

Since the insect sensory system directly reads its external environment, the AP appears as a powerful tool to study habitat adaptation [83,124], host-searching behavior, and dispersal ability [122], or its possible association with insecticide resistance [61] or with parasitism [125]. Other interesting points inviting to study antennae are that (i) they can be explored in nymphs, (ii) they provide valuable information of sexual differentiation, and (iii) as bilateral structures, antennae could be used to explore the developmental stability of the insect (see Section 2.5.2).

## 4. Discussion

### 4.1. The Measurement Error

Measurement error is an often neglected chapter in published studies, at the risk of leading to classification errors [126,127]. Measurement error exists at different stages of morphometric analysis: the mounting technique of specimens or organs, the photography conditions, and the user’s skill in collecting landmark coordinates [128]. In general, modern digital photography techniques provide adequate resolution for correct landmark recognition under different conditions. However, it may be convenient to photograph a distortion test chart to ensure that there is no image distortion. Among the sources of measurement error other than those resulting from an incorrect photography step, user intervention is often the most important. The error is usually due to small but systematic differences between different users in the exact location of the same landmarks. These subtle discrepancies can be seriously amplified by the power of a classification analysis such as discriminant analysis [129]. Measurement error can become a significant obstacle to mixing data from different users to enable a larger sample study [129,130]. As a result, User A should not enter their own measurements into a coordinate database collected by User B, and vice versa.

Currently, the recommended method for performing morphometric comparisons is to allow a single user to produce the data (see next paragraph). Even when performed by a single user, the digitization step should be repeated at least once, allowing its accuracy to be measured and error to be reduced by averaging the two digitizations [128].

### 4.2. Distinguishing Species by Shape

Significant differences in geometric shape between populations, even if free of allometric effects (see Section 2.2.3), should not be the sole argument for deciding the specific status of populations. In fact, no new species of Triatominae have been declared on the basis of morphometric variation alone. Studies describing new species [131] or exploring species homogeneity have generally used morphometric variation as a complementary argument [35,132,133]. Because of the difficulty of clearly defining what a species is, the modern trend in taxonomy is to combine various disciplines to help reach consensus on the specific status of new or questioned taxa [6], an approach now called integrative taxonomy [134,135]. However, thanks to a simple morphometric analysis, certain taxa have been legitimately called into question [136], synonymized [137,138], detected [139], or even provisionally explained [140].

In other insect groups, such as Diptera, geometric shape variation has proven to be a reliable character for detecting or distinguishing morphologically close, cryptic, or even sibling species [23,28], and there is no evidence that this would be different in Triatominae. Morphometric studies based on geometric shape have distinguished cryptic species such as *R. prolixus* and *R. robustus* [141,142,143], or morphologically close species within the *R. prolixus* complex (11 species) [99], within the *T. brasiliensis* complex [97], within the *T. oliverai* complex [144], within the *T. sordida* subcomplex [98,145], or evolutionary lineages within the *T. dimidiata* complex [27,36] (but see [82]) or the *T. pallidipennis* complex [38]. Most of the papers demonstrating the usefulness of the shape variable in species recognition have been applied to adults. However, its application to juvenile stages has been successfully tested (see Section 3.1 and Section 3.2) and could be of high epidemiological interest because the presence of pre-adult stages in the home may suggest the ability of the species to colonize human habitations, an important condition for being a vector.

### 4.3. Phylogeny?

After a classic morphometric study presenting the level of separation of certain species based on morphometric traits, the temptation is never far away to make a phylogenetic presentation. However, there is no clear basis for expecting parallelism between the phylogenetic signal of shape variables and that of genetic markers [22]. According to theoretical and modelization studies, the phylogenetic signal embedded in landmark locations is not strong enough to provide reliable results [146,147].

And yet, even a simple UPGMA tree derived from the wing venation architecture could be in global agreement with phylogenetic trees derived from genetic characters: a striking example is the clear proximity of the New World wings shapes with the North American ones [37]. Another example is the inclusion of a predator, *Opisthacidius pertinax*, in the UPGMA tree derived from the wing shape of Triatominae [148], as confirmed later in molecular phylogenetic trees [149]. The above-mentioned examples confirm that divergence of form between species contains the effects of large evolutionary events, without, however, reflecting these events alone. Indeed, other examples of phylogenetic hypotheses drawn from either geometric shape [33,96,97,118,144,150] or shape as derived from linear measurements [144,151,152] do not always show complete congruence with related molecular trees.

In fact, the morphogenetic processes underlying morphological changes are not supposed to ensure a linear reflection of genetic changes, which makes morphometric changes unpredictable indicators of underlying genetic changes. In conclusion, to examine the relationship between shape variation and evolutionary events, it is less questionable (and less risky) to fit shape data to a known phylogeny than to estimate a phylogeny from shape data [22,146].

### 4.4. Exploring Species Homogeneity

Morphometric variation within a species has generally been explored between pre-established groups, such as geographical areas (Section 4.4.1), color varieties (Section 4.4.2), or habitats (Section 4.5.1). Another approach is to explore the structure of a population without apparent subdivision (Section 4.5.2) with its important epidemiological correlate: the probability of reinfestations after control measures (Section 4.5.3).

#### 4.4.1. Geographic Variation

Large-scale geographic studies of populations of genera *Triatoma* and *Rhodnius* observed a progressive reduction in body size from the putative geographic origin and the periphery of the species’ range. In *T. infestans*, the decrease in body size was accompanied by a reduction in chromosome heterochromatin blocks [27,153]. Based on these observations in *T. infestans*, new hypotheses were suggested for *R. prolixus* from Central America. Indeed, the analyses in this region showed a decrease in the size of the head of *R. prolixus*, as well as a significantly lower number of RAPD bands relative to Venezuelian populations. In agreement with historical data, these clines suggest that the Central American populations represents a recently imported population, therefore, in principle, easier to eliminate [154].

Morphometric properties (size and shape) using either linear measurements [155] or landmark-based methods are frequently applied with the idea to detect or to reinforce the hypothesis of microevolutionary events in *T. infestans* [55,156], *T. garciabesi* [39,56], *T. guasayana* [56,86,87], *T. nitida* [155] or *Rhodnius ecuadoriensis* [109]. Such studies are always a source of information about the within-species microevolutionary trends, and can detect or suspect possible speciation events like in *Panstrongylus rufotuberculatus* of Bolivia [133], in *R. pallescens* of Colombia and Panama [157], in *T. sordida* [158], *T. patagonica* [159], *T. dimidiata* [27,36], or *T. pallidipennis* [38]. In addition to detection of possible speciation processes, the within-species geographic variation of morphometric properties also has important epidemiological applications (see Section 4.5.2).

#### 4.4.2. Chromatic Forms

Intuitively, morphometric variation should parallel morphological variation. However, between *T. infestans* and *T. melanosoma*, there is a clear lack of parallelism between chromatic variation and wing shape [138], or between chromatic variation and antennal phenotype [137], sufficient to question the validity of *T. melanosoma* as a distinct species from *T. infestans*. In Bolivia, no morphometric evidence was found to consider the “dark morph” as a distinct species from *T. infestans* [160]. In Argentina, chromatic and non-chromatic morphs of *T. infestans* showed significant differences in size and shape but did not question their conspecificity [55].

Shape differences between different conspecific forms are interesting to know from a taxonomic point of view, but they do not tell the whole story. In the morphometrist’s toolbox, there are other ways to understand local morphological variation, such as symmetry (see Section 2.5.2) or sexual dimorphism (see Section 2.5.1), not to mention antennal phenotype (see Section 3.4). Consider, for example, the chromatic and non-chromatic forms of species *T. infestans* in domestic and peridomestic habitats of Argentina. They show similar sexual dimorphism in size, but they display different levels of asymmetry: higher in melanic individuals, suggesting a selective advantage of non-melanic individuals in domestic and peridomestic habitats [55].

### 4.5. Targeting Bugs

The target of a vector control programme must be properly delimited at different geographical scales and, in addition to the choice of effective insecticides, must be prepared for possible reinfestation problems. In this respect, the morphometric approach has been useful.

#### 4.5.1. Domestic Versus Peridomestic or Sylvatic Populations

The apparent distinction between domestic and peridomestic populations [155], or sylvatic [161], of the same species is often examined because of its epidemiological importance. Are these isolated populations or are they connected, jeopardizing habitat control measures [162,163]?

It has been suggested that the size of kissing bugs decreases during the transition from sylvatic or peridomestic to domestic foci of *T. infestans* [70,71]. This transition can be seen as a shift from low to high population density, but also from a wild to an artificial environment. Furthermore, insect size also exhibits seasonal variation that may affect this pattern [101]. To explore this question, morphometric differences between a field population and its laboratory descendants were presented as a model for the changes observed between natural samples of sylvatic and domestic populations.

In *Panstrongylus geniculatus*, the analyses over five generations showed differences in size but not in shape, with the decrease in head size occurring only up to the second generation while the decrease in wing size proceeded up to the fifth generation [164]. A laboratory protocol in *R. pallescens* allowed to reproduce the reduction in size in the first laboratory generation [54] by simulating the apparent changes occurring for a Triatominae in the transition from silvatic (low population density, low feeding frequency) to domestic habitats (higher density, higher feeding frequency). As in *P. geniculatus*, size decreased but no shape differences were observed other than a passive change in shape with size change. The authors emphasized the importance of the interaction between population density and feeding frequency in producing specific and significant changes in insect dimensions [54].

In *R. neglectus*, a reduction in wing size was observed between sylvatic and laboratory conditions [165]. A similar pattern of size reduction was observed in Brazilian *T. infestans* samples [166]. All these observations and experiments support the idea that size changes can be used as a marker of the colonization of human habitations by Triatominae, as recently confirmed in species *T. sordida* from Brazil [167].

To help distinguishing domestic from peridomestic or sylvatic specimens, another morphometric strategy was explored: the sexual dimorphism (see Section 2.5.1). A reduction in sexual dimorphism has been suggested as a signal of established domestication [70,71,165]. This suggestion was frequently verified, to the extent of considering it as an argument to infer the recentness of the domestic populations of *T. venosa* [168].

Another very different morphometric strategy was also suggested to compare domestic and field samples of Triatominae: the measurement of symmetry. A significant asymmetry in the wings of domestic samples was apparent in *T. sordida* from Bolivia, while it was not detectable in its field counterparts [71]. Later studies on Triatominae also suggested the level of asymmetry (actually of the fluctuating asymmetry) as a possible marker of different factors, all related to possible sources of stress, either genetic or environmental ones (see Section 2.5.2).

As already mentioned above (see Section 3.4), the sensory pattern of antennal segments also contributed to characterize sylvatic and (peri)domestic populations [108,124,125].

#### 4.5.2. Biogeographical Islands

Exploring morphometric variation within a species at a broader spatial scale could help design control strategies by detecting isolation, i.e., the absence of exchanges of individuals, between certain territories that would therefore behave like isolated “islands”. In this situation, vector control measures could be applied sequentially, one area (“island”) after another.

It seems commonly admitted that the adaptation to different ecotopes or microhabitats (ecological pressure) is perhaps the main mechanism that drives the speciation in the subfamily Triatominae [27,71,109]. This adaptation could generate morphological changes, generally qualitative, even before the installation of reproductive isolation [109,169]. But before developing these rapid morphological changes (see Section 4.4.1), the separated populations develop a less visible, but significant, morphometric divergence.

Thus, from the point of view of the morphometrician, but also from the point of view of the epidemiologist, it is necessary to distinguish reproductive isolation from physical isolation (geographical, mainly). Shape characters, in particular the internal wing venation architecture, diverge first because of spatial isolation (genetic drift, see Section 2.2.2). In fact, between isolated conspecific populations, the internal wing venation architecture would be affected because of both genetic drift and environmental factors [56,87,109,170]. In the case of gene exchanges between populations, even allopatric ones, or at the frontier between two biogeographical islands [39], no genetic drift would take place and the internal wing venation architecture would hardly diverge. My working hypothesis has a corollary: in a common environment, the venation architecture diverges between spatially isolated subpopulations, even at a very low spatial scale [100,156,171]. In another insect, *Aedes aegypti*, two isolated isofemale laboratory lines, in spite of a common laboratory environment, could diverge in the geometry of wing venation after less than fifteen generations [172]. Intraspecific studies could help vector control strategy by detecting biogeographical islands, or it could help to understand the origin of reinfestations after control measures (see Section 4.5.3) [28].

#### 4.5.3. Reinfestation Studies

As for linear morphometric measurements [9], the geometric method was applied to the recurring problem of the origin of reinfesting specimens after control measures: In the absence of insecticide resistance, do reinfesting individuals come from a residual population or from untreated neighboring foci? In order to try to answer this question using shape variables or using the antennal phenotype, a few conditions are necessary.

First, the target sample (the pre-treatment population) and neighboring samples must be morphometrically distinguishable [161], at a small [83,100,124,156,173] or at a larger geographic scale [83,108,158,174]. Second, it is assumed that in such a comparison, unexpected, cryptic species would be detected, such as *R. robustus* and *R. prolixus* [175], *R. neglectus* and *R. prolixus* [142], and others.

Furthermore, as this type of study [163,176,177,178] can compare several generations of insects (before and after control measures), it should be clear to what extent the morphometric shape is transmitted to subsequent generations [162] and to what extent the geometric shape changes over time [101,166,179,180].

Finally, as the statistical procedure consists in assigning a sample (reinfested specimen) to several populations of different generations, the interference of a possible correct prediction by mere chance must be taken into account (see *TAU* statistics in Section 2.4).

### 4.6. Vector Biology

#### 4.6.1. Feeding Habits

Triatominae are obligate blood-sucking insects, with nymphs, males, and females all feeding on vertebrate blood. Exceptional or occasional predatory behavior has been reported, suggesting their evolutionary origin as predators [181]. Experimental work compared the effect of bird and mammalian (pigeon, guinea pig) blood sources on the development of *T. infestans*, showing significant effects on head shape and size [60,105]. Later experiments confirmed that different blood sources can have different effects on head or body shape in other species, as in *T. williami* [106] or in *T. pallidipennis* [79].

#### 4.6.2. Dispersal Capacity

Dispersal of bugs is possible through flight and walking [92]. When it comes to flight, morphometric studies of Triatominae have not necessarily focused on wings: other aspects of bug morphology have been associated with different flight abilities [102,103].

When comparing wings of natural populations, it must be specified which wing shape is being compared (see Section 3.3.2), but this does not constitute sufficient precision. While not omitting the caveat regarding statistical association and causality, other obstacles inherent in studies of natural populations must also be considered.

(i) To the possible exception of laboratory experiments, there is frequently an important additional effect modifying size and shape between categories: geography or simply spatial isolation. Some studies exploring the flight ability of the bugs are at the same time interesting studies about the geographic variation of metric properties [56,86,87]. In the case of both allopatry and distinct environments (urban, rural, etc.), it might become very difficult to disentangle these interfering factors. An example of a possible solution uses the optimum K of the Kmeans approach and the metric disparities of various samples configurations [61].

(ii) In addition to variation in shape between populations adapted to different environments or microenvironments, variation in size (not just wing size) is frequently observed. Since in such comparisons size often shows significant differences, shape comparisons should take care to be free of allometry (see Section 2.2.3). If this is not possible (or not required, as in [80]), the effect of size on shape should be taken into account in the overall interpretation, or its importance should be illustrated graphically (as in [182]).

There is one morphometric trait that may help to circumvent at least some of these obstacles: insect symmetry (see Section 2.5.2). As an indicator of developmental instability, symmetry may be more related to the environment than to genetic drift. The relationship between increased asymmetry and reduced flight ability was already suspected in a simple laboratory experiment where flight ability was assessed by flight activity [81]. More comprehensive studies have been published recently [55,78,80,179].

#### 4.6.3. Insecticide Susceptibility

This important aspect of insect physiology was approached by tentatively answering two questions: (i) Is there an association between the application of sublethal doses of pyrethroid insecticides and the metric properties of insects [77,78,170]? and (ii) Are resistant specimens detectable morphometrically [61]?

Variation in the fluctuating asymmetry of wings of *T. infestans* collected before and after a community-wide pyrethroid spraying campaign in Argentina suggested the possible effects of a lower population density after spraying or a selective advantage of more symmetrical individuals [77]. A laboratory experiment on *T. infestans* concluded that insects exposed to a sublethal dose of deltamethrin had larger, less symmetrical, and less canalized wings [78]. In another laboratory experiment comparing pyrethroid-sensitive and pyrethroid-resistant *T. infestans*, insects previously treated with deltamethrin developed significantly thicker cuticles than the control only in the resistant population and significantly larger wings in both populations [170].

Recently, the morphometric properties of natural populations of resistant *T. infestans* specimens were examined for their wings, heads, and antennae. Various differences were suspected to be specific of insecticide resistance acquisition, including smaller size of the wings and heads, shape differences, and antennal sensory simplification, laying the foundations for the identification of a resistance biomarker in Triatominae [61].

## 5. Conclusions

Over the last three decades, the morphometric study of Chagas disease vectors has undergone considerable development and a steady increase in the quality of publications. In this group of vectors, morphometrics has been successfully applied not only to clarify species delimitation issues, but also, and perhaps more importantly, to reveal and understand their intraspecific variation. In this way, modern morphometrics applied to Triatominae has maintained a balance between two objectives: to detect and understand the microevolutionary (and beyond) events of an insect and to provide useful information for the development of vector control strategies.

## References

[1] Chagas C. (1909). Nova tripanosomiase humana. Gaz. Medica Bahia.

[2] Mougabure-Cueto G., Picollo M.I. (2015). Insecticide resistance in vector Chagas disease: Evolution, mechanisms and management. Acta Trop..

[3] Cavallo M.J., Amelotti I., Gorla D.E. (2016). Invasion of rural houses by wild Triatominae in the arid Chaco. J. Vector Ecol..

[4] Dujardin J.P., Schofield C.J., Tibayrenc M. (2007). Vector control by surveillance networks: The ECLAT program and Chagas. Encyclopedia of Infectious Diseases: Modern Methodologies.

[5] Schofield C.J., Diotaiuti L., Dujardin J.P. (1999). The process of Domestication in Triatominae. Mem. Inst. Oswaldo Cruz.

[6] Bargues M., Schofield C., Dujardin J.P., Telleria J., Tibayrenc M. (2017). 6-Classification and systematics of the Triatominae. American Trypanosomiasis Chagas Disease.

[7] de Paiva V.F., Belintani T., de Oliveira J., Galvão C., da Rosa J.A. (2022). A review of the taxonomy and biology of Triatominae subspecies (Hemiptera: Reduviidae). Parasitol. Res..

[8] Noireau F., Dujardin J.P., Telleria J., Tibayrenc M. (2010). Biology of Triatominae. American Trypanosomiasis. Chagas Disease. One Hundred Years of Research.

[9] Dujardin J.P., Bermúdez H., Schofield C.J. (1997). The use of morphometrics in entomological surveillance of silvatic foci of *Triatoma infestans* in Bolivia. Acta Trop..

[10] Rohlf F.J., Marcus L.F. (1993). A revolution in morphometrics. TREE.

[11] Lent H., Wygodzinsky P. (1979). Revision of the Triatominae (Hemiptera, Reduviidae), and their significance as vectors of Chagas disease. Bull. Am. Mus. Nat. Hist..

[12] Sneath P., Sokal R. (1962). Numerical taxonomy. Nature.

[13] Gorla D.E., Jurberg J., Catalá S.S., Schofield C.J. (1993). Systematics of Triatoma sordida, T. guasayana and T. patagonica (Hemiptera, Reduviidae).

[14] Harry M. (1994). Morphometric variability in the Chagas’ disease vector *Rhodnius prolixus*. Jpn. J. Genet..

[15] Torres E., Galíndez G., Itamar M.E., Araujo V., Barazarte R., Marquez J. (1997). Multivariate analysis applied to some populations of *Panstrongylus geniculatus* Latreill 1811 (Hemiptera, Reduviidae, Triatominae). Entomol. Vect..

[16] Dujardin J.P., Forgues G., Torrez M., Martínez E., Cordoba C., Gianella A. (1998). Morphometrics of domestic *Panstrongylus rufotuberculatus* in Bolivia. Ann. Trop. Med. Parasitol..

[17] Rohlf F.J. (1971). Perspectives on the application of multivariate statistics to taxonomy. Taxon.

[18] Klingenberg C.P., Marcus L.F., Corti M., Loy A., Naylor G.J.P., Slice D. (1996). Multivariate allometry. Advances in Morphometrics Proceedings of the 1993 NATO-ASI on Morphometrics.

[19] Rohlf F.J., Archie J.W. (1984). A comparison of Fourier methods for the description of wing shape in mosquitoes (Diptera:Culicidae). Syst. Zool..

[20] Rohlf F.J., Rohlf F., Bookstein F. (1990). Rotational fit (Procrustes) methods. Proceedings of the Michigan Morphometrics Workshop.

[21] Bookstein F.L. (1991). Morphometric Tools for Landmark Data. Geometry and Biology.

[22] Rohlf F.J. (2000). Geometric Morphometrics in Systematics. https://api.semanticscholar.org/CorpusID:53362120.

[23] Dujardin J.P., Slice D.E., Tibayrenc M. (2006). Contributions of Morphometrics to Medical Entomology. Encyclopedia of Infectious Diseases.

[24] Dujardin J.P. (2008). Morphometrics applied to Medical Entomology. Infect. Genet. Evol..

[25] Perrard A., Baylac M., Carpenter J.M., Villemant C. (2014). Evolution of wing shape in hornets: Why is the wing venation efficient for species identification?. J. Evol. Biol..

[26] Zúñiga-Reinoso Á., Benítez H.A. (2015). The overrated use of the morphological cryptic species concept: An example with *Nyctelia* darkbeetles (Coleoptera: Tenebrionidae) using geometric morphometrics. Zool. Anz..

[27] Dujardin J.P., Costa J., Bustamante D., Jaramillo N., Catalá S. (2009). Deciphering morphology in Triatominae: The evolutionary signals. Acta Trop..

[28] Dujardin J.P., Tibayrenc M. (2024). Chapter 16—Modern Morphometrics of Arthropods: A Phenotypic Approach to Species Recognition and Population Structure. Genetics and Evolution of Infectious Diseases.

[29] Bookstein F.L., Rohlf F.J., Bookstein F.L. (1990). Introduction to methods for landmark data. Proceedings of the Michigan Morphometrics Workshop.

[30] Adams D.C. (1999). Methods for shape analysis of landmark data from articulated structures. Evol. Ecol. Res..

[31] Kuhl F.P., Giardina C.R. (1982). Elliptic Fourier features of a closed contour. Comput. Graph. Image Process..

[32] Bookstein F.L. (1997). Landmark methods for forms without landmarks: Localizing group differences in outline shape. Med. Image Anal..

[33] Santillán-Guayasamín S., Villacís A., Grijalva M., Dujardin J. (2017). The modern morphometric approach to identify eggs of Triatominae. Parasit Vectors.

[34] Santillan Guayasamin S., Villacis A.G., Grijalva M.J., Dujardin J.P. (2018). Triatominae: Does the shape change of non-viable eggs compromise species recognition?. Parasites Vectors.

[35] Villacís A., Dujardin J., Panzera F., Yumiseva C., Pita S., Santillán-Guayasamín S., Orozco M., Mosquera K., Grijalva M. (2020). Chagas vectors *Panstrongylus chinai* (Del Ponte, 1929) and *Panstrongylus howardi* (Neiva, 1911): Chromatic forms or true species?. Parasit Vectors.

[36] Cruz D., Arellano E., Denis Ávila D., Ibarra-Cerdena C.N. (2020). Identifying Chagas disease vectors using elliptic Fourier descriptors of body contour: A case for the cryptic dimidiata complex. Parasites Vectors.

[37] Dujardin J.P., Pham Thi K., Truong Xuan L., Panzera F., Pita S., Schofield C.J. (2015). Epidemiological status of kissing-bugs in South East Asia: A preliminary assessment. Acta Trop..

[38] Cruz D.D., Ospina-Garcés S.M., Arellano E., Ibarra-Cerdeña C.N., Nava-García E., Alcalá R. (2023). Geometric morphometrics and ecological niche modelling for delimitation of *Triatoma pallidipennis* (Hemiptera: Reduviidae: Triatominae) haplogroups. Curr. Res. Parasitol. Vector-Borne Dis..

[39] Verly T., Pita S., Carbajal de la Fuente A., Burgueño Rodríguez G., Piccinali R., Fiad F., Ríos N., Panzera F., Lobbia P., Sanchez Casaccia P. (2024). Relationship between genetic diversity and morpho-functional characteristics of flight-related traits in *Triatoma garciabesi* (Hemiptera: Reduviidae). Parasites Vectors.

[40] Dujardin J.P. (2019). A template-dependent semilandmarks treatment and its use in medical entomology. Infect. Genet. Evol..

[41] Rohlf F. (2015). The Tps series of software. Hystrix.

[42] Baken E., Collyer M., Kaliontzopoulou A., Adams D. (2021). geomorph v4.0 and gmShiny: Enhanced analytics and a new graphical interface for a comprehensive morphometric experience. Methods Ecol. Evol..

[43] Schlager S., Zheng G., Li S., Szekely G. (2017). Morpho and Rvcg—Shape Analysis in R. Statistical Shape and Deformation Analysis.

[44] Klingenberg C.P. (2011). MorphoJ: An integrated software package for geometric morphometrics. Mol. Ecol. Resour..

[45] Dujardin S., Dujardin J.P. (2019). Geometric morphometrics in the cloud. Infect. Genet. Evol..

[46] Bonhomme V., Picq S., Gaucherel C., Claude J. (2014). Momocs: Outline Analysis Using R. J. Stat. Softw..

[47] Iwata H., Ukai Y. (2002). A computer program package for quantitative evaluation of biological shapes based on elliptic Fourier descriptors. J. Hered..

[48] Klingenberg C.P., Leamy L.J. (2001). Quantitative genetics of geometric shape in the mouse mandible. Evolution.

[49] Klingenberg C.P., Leamy L.J., Cheverud J.M. (2004). Integration and modularity of quantitative trait locus effects on geometric shape in the mouse mandible. Genetics.

[50] Shrimpton A., Robertson A. (1988). The isolation of polygenic factors controlling bristle score in *Drosophila melanogaster*: I. Allocation of third chromosome bristle effects to chromosome sections. Genetics.

[51] Carbajal-de-la Fuente A., Piccinali R., Porcasi X., Marti G., Rojas de Arias A., Abrahan L., Suárez F., Lobbia P., Medina G., Provecho Y. (2024). Variety is the spice: The role of morphological variation of *Triatoma infestans* (Hemiptera, Reduviidae) at a macro-scale. Acta Trop..

[52] Dujardin J.P., Kaba D., Solano P., Dupraz M., McCoy K.D., Jaramillo-O N. (2014). Outline-based Morphometrics, an overlooked method in arthropod studies?. Infect. Genet. Evol..

[53] Bellman R. (1961). Adaptive Control Processes: A Guided Tour.

[54] Caro-Riaño H., Jaramillo N., Dujardin J.P. (2009). Growth changes in *Rhodnius pallescens* under simulated domestic and sylvatic conditions. Infect. Genet. Evol..

[55] Nattero J., Carbajal de la Fuente A.L., Piccinali R.V., Cardozo M., Rodríguez C.S., Crocco L.B. (2020). Characterization of melanic and non-melanic forms in domestic and peridomestic populations of *Triatoma infestans* (Hemiptera: Reduviidae). Parasites Vectors.

[56] Fiad F., Cardozo M., Nattero J., Gigena G.V. (2024). Gorla, D.; Rodríguez, C. Association between environmental gradient of anthropization and phenotypic plasticity in two species of triatomines. Parasites Vectors.

[57] Good P. (2000). Permutation Tests. A Practical Guide to Resampling Methods for Testing Hypotheses.

[58] Debat V., Begin M., Legout H., David J.R. (2003). Allometric and nonallometric components of *Drosophila* wing shape respond differently to developmental temperature. Evolution.

[59] Collyer M.C., Adams D.C. (2007). Analysis Of Two-State Multivariate Phenotypic Change In Ecological Studies. Ecology.

[60] Nattero J., Leonhard G., Gürtler R.E., Crocco L.B. (2015). Evidence of selection on phenotypic plasticity and cost of plasticity in response to host-feeding sources in the major Chagas disease vector *Triatoma infestans*. Acta Trop..

[61] Hernández M.L., Dujardin J.P., Villacís A.G., Yumiseva C.A., Remón C., Mougabure-Cueto G. (2023). Resistance to deltamethrin in *Triatoma infestans* (Hemiptera: Reduviidae): Does it influence the phenotype of antennae, wings, and heads?. Acta Trop..

[62] Mitteröcker P., Bookstein F. (2011). Linear discrimination, ordination, and the visualization of selection gradients in modern morphometrics. Evol. Biol..

[63] Bookstein F.L. (2019). Pathologies of Between-Groups Principal Components Analysis in Geometric Morphometrics. Evolutionary Biology.

[64] Pimentel R.A., Foottit R.G., Sorensen J.T. (1992). An introduction to ordination, principal components analysis and discriminant analysis. Ordination in the Study of Morphology, Evolution and Systematics of Insects: Applications and Quantitative Genetic Rationales.

[65] Dujardin J., Dujardin S., Kaba D., Santillán-Guayasamín S., Villacís A.G., Piyaselakul S., Sumruayphol S., Samung Y., Morales-Vargas R. (2017). The maximum likelihood identification method applied to insect morphometric data. Zool. Syst..

[66] Gurgel-Gonçalves. R., Komp E., Campbell L.P., Khalighifar A., Mellenbruch J., Mendonça V.J., Owens H.L., de la Cruz Felix K., Peterson A.T., Ramsey J.M. (2017). Automated identification of insect vectors of Chagas disease in Brazil and Mexico: The Virtual Vector Lab. PeerJ.

[67] de Miranda V.L., de Souza E.P., Bambil D., Khalighifar A., Peterson A.T., de Oliveira Nascimento F.A., Gurgel-Gonçalves R., Abad-Franch F. (2024). Cellphone picture-based, genus-level automated identification of Chagas disease vectors: Effects of picture orientation on the performance of five machine-learning algorithms. Ecol. Inform..

[68] Kitthawee S., Dujardin J. (2016). The *Diachasmimorpha longicaudata* complex in Thailand discriminated by its wing venation. Zoomorphology.

[69] Klecka W.R. (1980). Discriminant analysis. Sage University Paper Series on Quantitative Applications in the Social Sciences.

[70] Dujardin J.P., Steindel M., Chavez T., Martínez E., Schofield C.J. (1999). Changes in the sexual dimorphism of Triatominae in the transition from natural to artificial habitats. Memórias Inst. Oswaldo Cruz.

[71] Dujardin J.P., Panzera P., Schofield C.J. (1999). Triatominae as a model of morphological plasticity under ecological pressure. Memórias Inst. Oswaldo Cruz.

[72] Klingenberg C.P., McIntyre G. (1998). Geometric morphometrics of developmental instability: Analyzing patterns of fluctuating asymmetry with Procrustes methods. Evolution.

[73] Klingenberg C.P. (2015). Analyzing Fluctuating Asymmetry with Geometric Morphometrics: Concepts, Methods, and Applications. Symmetry.

[74] Claude J. (2008). Morphometrics with R.

[75] Palmer A.R., Strobeck C. (1986). Fluctuating Asymmetry: Measurement, Analysis, Patterns. Annu. Rev. Ecol. Syst..

[76] Van Dongen S.V. (2006). Fluctuating asymmetry and developmental instability in evolutionary biology: Past, present and future. J. Evol. Biol..

[77] Nattero J., Piccinali R.V., Gaspe M.S., Gürtler R.E. (2019). Fluctuating asymmetry and exposure to pyrethroid insecticides in *Triatoma infestans* populations in northeastern Argentina. Infect. Genet. Evol..

[78] Nattero J., Mougabure-Cueto G., Debat V., Gürtler R.E. (2021). Phenotypic plasticity, canalisation and developmental stability of *Triatoma infestans* wings: Effects of a sublethal application of a pyrethroid insecticide. Parasites Vectors.

[79] Gutiérrez-Cabrera A., Badillo R., Gonzalez L., Ospina-Garcés S., Córdoba-Aguilar A. (2022). Body shape and fluctuating asymmetry following different feeding sources and feeding time in a triatomine, *Triatoma pallidipennis* (Stal, 1892). Infect. Genet. Evol..

[80] Nattero J., Piccinali R., Fiad F., Cano F., Carbajal-de-la Fuente A. (2023). Relationship between flight muscle dimorphism and wing morphometry in *Triatoma infestans* (Klug, 1834) (Hemiptera, Reduviidae, Triatominae). Front. Ecol. Evol..

[81] Soares R., Dujardin J.P., Schofield C., Diotaiuti L. (1996). Wing symmetry and flight activity. Mem. Inst. Oswaldo Cruz.

[82] Nouvellet P., Ramirez-Sierra M.J., Dumonteil E., Gourbière S. (2011). Effects of genetic factors and infection status on wing morphology of *Triatoma dimidiata* species complex in the Yucatán peninsula, Mexico. Infect. Genet. Evol..

[83] Hernández M.L., Abrahan L.B., Dujardin J.P., Gorla D.E., Catalá S.S. (2011). Phenotypic Variability and Population Structure of Peridomestic *Triatoma infestans* in Rural Areas of the Arid Chaco (Western Argentina): Spatial Influence of Macro- and Microhabitats. Vector Borne Zoonotic Dis..

[84] Rohlf F.J., Corti M. (2000). Use of two-block partial least-squares to study covariation in shape. Syst. Biol..

[85] Escoufier Y. (1973). La dépendance de deux aléas vectoriels critères et visualisation. Rev. Stat. Appliquée.

[86] Gigena V., Rodríguez C., Fiad F., Hernández M., Carbajal-de-la Fuente A., Piccinali R., Casaccia P., Rojas de Arias A.R., Lobbia P., Abrahan L. (2023). Phenotypic variability in traits related to flight dispersal in the wing dimorphic species *Triatoma guasayana*. Parasit Vectors.

[87] Fiad F.G., Cardozo M., Rodríguez C.S., Hernández M.L., Crocco L.B., Gorla D.E. (2022). Ecomorphological variation of the *Triatoma guasayana* wing shape in semi-arid Chaco region. Acta Trop..

[88] Barata J.M.S. (1981). Aspectos morfológicos de ovos de Triatominae. II. Caracteristicas macroscopicas e exocoriais de dez especies do gênero *Rhodnius* Stal, 1859 (Hemiptera, Reduviidae). Rev. Saúde Publica.

[89] Costa J., Barth O.M., Marchon Silva V., de Almeida C.E., FreitasSibajev M.G.R., Panzera F. (1997). Morphological studies on the *Triatoma brasiliensis* Neiva, 1911 (Hemiptera, Reduviidae, Triatominae) genital structures and eggs of different chromatic forms. Memórias Inst. Oswaldo Cruz.

[90] Souza S. (2011). Aspectos Morfológicos e Morfometria de Ovos de Triatoma pseudomaculata Corrêa & Espínola, 1964 (Hemiptera, Reduviidae, Triatominae) No Estado do Ceará.

[91] Obara M.T., Barata J., da Silva N.N., Ceretti Júnior W., Urbinatti P.R., da Rosa J.A., Jurberg J., Galvão C. (2007). Morphologic, morphometrical, and histological aspects of the eggs of four species of the genera *Meccus* (Hemiptera, Reduviidae, Triatominae). Mem. Inst. Oswaldo Cruz.

[92] Abrahan L.B., Gorla D.E., Catalá S.S. (2011). Dispersal of *Triatoma infestans* and other Triatominae species in the arid Chaco of Argentina: Flying, walking or passive carriage? The importance of walking females. Mem. Inst. Oswaldo Cruz.

[93] Patterson J.S., Barbosa S.E., Feliciangeli M.D. (2009). On the genus *Panstrongylus* Berg 1879: Evolution, ecology and epidemiological significance. Acta Trop..

[94] Rocha D.S., Patterson J.S., Sandoval C.M., Jurberg J., Ângulo V.M., Esteban L., Galvão C. (2005). Description and ontogenetic morphometrics of nymphs of *Belminus herreri* Lent & Wygodzinsky (Hemiptera: Reduviidae, Triatominae). Neotrop. Entomol..

[95] Rocha D., Dale C., da Rosa J.A., Galvão C. (2020). Description of nymphs and ontogenetic morphometry of *Triatoma ryckmani* Zeledón & Ponce, 1972 (Hemiptera: Heteroptera: Reduviidae: Triatominae). EntomoBrasilis.

[96] Lázari Cacini G., de Oliveira J., Belintani T., dos Santos Souza E., Olaia N., Pinto M.C., da Rosa J. (2022). Immature instars of three species of *Rhodnius* Stȧl, 1859 (Hemiptera, Reduviidae, Triatominae): Morphology, morphometry, and taxonomic implications. Parasites Vectors.

[97] Oliveira J., Marcet P.L., Takiya D.M., Mendonça V.J., Belintani T., Bargues M.D., Mateo L., Chagas V., Folly-Ramos E., Cordeiro-Estrela P. (2017). Combined phylogenetic and morphometric information to delimit and unify the *Triatoma brasiliensis* species complex and the Brasiliensis subcomplex. Acta Trop..

[98] Nattero J., Piccinali R., Macedo Lopes C., Hernández M., Abrahan L., Lobbia P., Rodríguez C., Carbajal De La Fuente A. (2017). Morphometric variability among the species of the Sordida subcomplex (Hemiptera: Reduviidae: Triatominae): Evidence for differentiation across the distribution range of *Triatoma sordida*. Parasites Vectors.

[99] Alvarez A., Dale C., Galvão C. (2024). Geometric morphometry of the *Rhodnius prolixus* complex (Hemiptera, Triatominae): Patterns of intraspecific and interspecific allometry and their taxonomic implications. ZooKeys.

[100] Schachter-Broide J.S., Dujardin J.P., Kitron U., Gürtler R.E. (2004). Spatial structuring of *Triatoma infestans* (Hemiptera: Reduviidae) populations from northwestern Argentina using wing geometric morphometry. J. Med. Entomol..

[101] Schachter-Broide J., Gürtler R.E., Kitron U., Dujardin J.P. (2009). Temporal variations of wing size and shape of *Triatoma infestans* (Hemiptera: Reduviidae) populations from northwestern Argentina using geometric morphometry. J. Med. Entomol..

[102] Hernández M., Espinoza J., Gomez M., Gorla D. (2020). Morphological changes associated with brachypterous *Triatoma guasayana* (Hemiptera, Reduvii- dae) and their relationship with flight. Int. J. Trop. Insect Sci..

[103] Hernández M., Dujardin J., Gorla D., Catalá S. (2015). Can body traits, other than wings, reflect the flight ability of triatominae bugs?. Rev. Soc. Bras. Med. Trop..

[104] Grodnitsky D.L. (2000). Form and Function of Insect Wings: The Evolution of Biological Structures.

[105] Nattero J., Malerba R., Rodríguez C., Crocco L. (2013). Phenotypic plasticity in response to food source in *Triatoma infestans* (Klug, 1834) (Hemiptera, Reduviidae: Triatominae). Infect. Genet. Evol..

[106] Lunardi R.R., Benítez H.A., Câmara T.P., Gomes L.P., Arrais-Silva W.W. (2017). Head shape variation in response to diet in *Triatoma williami* (Hemiptera, Reduviidae: Triatominae), a possible Chagas disease vector of legal Amazonia. Zool. Anz..

[107] Vilaseca C., Méndez M.A., Pinto C.F., Lemic D., Benítez H.A. (2021). Unraveling the Morphological Variation of *Triatoma infestans* in the Peridomestic Habitats of Chuquisaca Bolivia: A Geometric Morphometric Approach. Insects.

[108] Catalá S., Dujardin J.P. (2001). Antennal sensilla patterns indicate geographic and ecotopic variability among *Triatoma infestans* (Hemiptera: Reduviidae) populations. J. Med. Entomol..

[109] Abad-Franch F., Monteiro F.A., Pavan M.G., Patterson J.S., Bargues M.D., Zuriaga M., Aguilar M., Beard C.B., Mas-Coma S., Miles M.A. (2021). Under pressure: Phenotypic divergence and convergence associated with microhabitat adaptations in Triatominae. Parasites Vectors.

[110] Dorn P.L., Justi S.A., Dale C., Stevens L., Galvão C., Lima-Cordón R., Monroy C. (2018). Description of *Triatoma mopan sp. n.* from a cave in Belize (Hemiptera, Reduviidae, Triatominae). ZooKeys.

[111] Monroy M.C., Bustamante D.M., Rodas A.G., Enriquez M.E., Rosales R.G. (2003). Habitats, dispersion and invasion of sylvatic *Triatoma dimidiata* (Hemiptera: Reduviidae: Triatominae) in Peten, Guatemala. J. Med. Entomol..

[112] Bustamante D.M., Monroy C., Menes M., Rodas A., Salazar-Schettino P.M., Rojas G., Pinto N., Guhl F., Dujardin J.P. (2004). Metric Variation among Geographic Populations of the Chagas Vector *Triatoma dimidiata* (Hemiptera: Reduviidae: Triatominae) and Related Species. J. Med. Entomol..

[113] de la Rúa N.M., Bustamante D.M., Menes M., Stevens L., Monroy C., Kilpatrick C.W., Rizzo D., Klotz S.A., Schmidt J., Axen H.J. (2014). Towards a phylogenetic approach to the composition of species complexes in the North and Central American *Triatoma*, vectors of Chagas disease. Infect. Genet. Evol..

[114] Gurgel-Gonçalves R., Ferreira J., Rosa A., Bar M., Galvão C. (2011). Geometric morphometrics and ecological niche modelling for delimitation of near-sibling triatomine species. Med. Vet. Entomol..

[115] Lima-Cordón R.A., Monroy M.C., Stevens L., Rodas A., Rodas G.A., Dorn P.L., Justi S.A. (2019). Description of *Triatoma huehuetenanguensis sp. n.*, a potential Chagas disease vector (Hemiptera, Reduviidae, Triatominae). ZooKeys.

[116] Almeida C.E., Oliveira H.L., Correia N., Dornak L.L., Gumiel M., Neiva V.L., Harry M., Mendonça V.J., Costa J., Galvão C. (2012). Dispersion capacity of *Triatoma sherlocki*, *Triatoma juazeirensis* and laboratory-bred hybrids. Acta Trop..

[117] Mougabure-Cueto G., Hernández M.L., Gilardoni J.J., Nattero J. (2024). Morphometric study of the legs of the main Chagas vector, *Triatoma infestans* (Hemiptera: Reduviidae). Acta Trop..

[118] Belintani T., de Paiva V.F., de Oliveira J., da Rosa J.A. (2022). New in morphometry: Geometric morphometry of the external female genitalia of Triatominae (Hemiptera: Reduviidae). Acta Trop..

[119] Catalá S., Schofield C.J. (1994). Antennal sensilla of *Rhodnius*. J. Morphol..

[120] Catalá S. (1997). Antennal sensilla of Triatominae. A comparative study of five genera. Int. J. Insect Morphol. Embryol..

[121] Gracco M., Catalá S.S. (2000). Inter-specific and developmental differences on the array of antennal chemoreceptors in four species of Triatominae (Hemiptera: Reduviidae). Mem. Inst. Oswaldo Cruz.

[122] May-Concha I., Guerenstein P.G., Ramsey J.M., Rojas J.C., Catalá S. (2016). Antennal phenotype of Mexican haplogroups of the *Triatoma dimidiata* complex, vectors of Chagas disease. Infect. Genet. Evol..

[123] Hernández M.L., Abrahan L., Moreno M., Gorla D., Catalá S. (2008). Phenotypic Variability Associated to Genomic Changes in the main vector of Chagas Disease in South America. Acta Trop..

[124] Abrahan L., Hernández L., Gorla D., Catalá S.S. (2008). Phenotypic diversity of *Triatoma infestans* at the microgeographic level on the Gran Chaco of Argentina and the Andean valleys of Bolivia. J. Med. Entomol..

[125] May-Concha I.J., Escalante-Talavera M.J., Dujardin J.P., Waleckx E. (2022). Does *Trypanosoma cruzi* (Chagas, 1909) (Kinetoplastida: Trypanosomatidae) modify the antennal phenotype of *Triatoma dimidiata* (Latreille, 1811) (Hemiptera: Triatominae)?. Parasites Vectors.

[126] Rasmussen P., Wheeler W., Moser T., Vine L., Sullivan B., Rusch D. (2001). Measurements of Canada goose morphology - Sources of error and effects on classification of subspecies. J. Wildl. Manag..

[127] Jordaens K., Van Dongen S., Van Riel P., Geenen S., Verhagen R., Backeljau T. (2002). Multivariate morphometrics of soft body parts in terrestrial slugs: Comparison between two datasets, error assessment and taxonomic implications. Biol. J. Linn. Soc..

[128] Arnqvist G., Mårtensson T. (1998). Measurement error in geometric morphometrics: Empirical strategies to assess and reduce its impact on measure of shape. Acta Zool. Acad. Sci. Hung..

[129] Dujardin J.P., Kaba D., Henry A.B. (2010). The exchangeability of shape. BMC Res. Notes.

[130] Robinson C., Terhune C.E. (2017). Error in geometric morphometric data collection: Combining data from multiple sources. Am. J. Phys. Anthropol..

[131] Galvao C., Patterson J.S., Rocha D.D., Jurberg J., Carcavallo R., Rajen K., Ambrose D.P., Miles M.A. (2002). A new species of Triatominae from Tamil Nadu, India. Med. Vet. Entomol..

[132] Mendonça V., Alevi K., Pinotti H., Gurgel-Gonçalves R., Pita S., Guerra A., Panzera F., Araújo R., Azeredo-Oliveir M., da Rosa J. (2016). Revalidation of *Triatoma bahiensis* Sherlock & Serafim, 1967 (Hemiptera: Reduviidae) and phylogeny of the *T. brasiliensis* species complex. Zootaxa.

[133] Pita S., Gómez-Palacio A., Lorite P., Dujardin J., Chavez T., Villacís A., Galvão C., Panzera Y., Calleros L., Pereyra-Mello S. (2022). Multidisciplinary approach detects speciation within the kissing bug *Panstrongylus rufotuberculatus* populations (Hemiptera, Heteroptera, Reduviidae). Mem. Inst. Oswaldo Cruz.

[134] Alevi K.C.C., de Oliveira J., Garcia A.C.C., Cristal D.C., Delgado L.M.G., de Freitas Bittinelli I., Dos Reis Y.V., Ravazi A., de Oliveira A.B.B., Galvão C. (2020). *Triatoma rosai* sp. nov. (Hemiptera, Triatominae): A New Species of *Argentinian Chagas* Disease Vector Described Based on Integrative Taxonomy. Insects.

[135] Alevi K.C.C., de Oliveira J., da Silva Rocha D., Galvão C. (2021). Trends in Taxonomy of Chagas Disease Vectors (Hemiptera, Reduviidae, Triatominae): From Linnaean to Integrative Taxonomy. Pathogens.

[136] Carbajal de la Fuente A.L., Jaramillo N., Barata J.M.S., Noireau F., Diotaiuti L. (2011). Misidentification of two Brazilian triatomes, *Triatoma arthurneivai* and *Triatoma wygodzinskyi*, revealed by geometric morphometrics. Med. Vet. Entomol..

[137] Catalá S., Torres M. (2001). Similitude of the patterns of sensilla on the antennae of *Triatoma melanosoma* and *Triatoma infestans*. Ann. Trop. Med. Parasitol..

[138] Gumiel M., Catalá S., Noireau F., de Arias A.R., Garcia A., Dujardin J.P. (2003). Wing geometry in *Triatoma infestans* (Klug) and *T. melanosoma* Martinez, Olmedo and Carcavallo (Hemiptera: Reduviidae). Syst. Entomol..

[139] Matias A., De la Riva J.X., Torrez M., Dujardin J.P. (2001). *Rhodnius robustus* in Bolivia identified by its wings. Mem. Inst. Oswaldo Cruz.

[140] Costa J., Peterson A.T., Dujardin J.P. (2008). Indirect evidences suggest homoploid hybridization as a possible mode of speciation in Triatominae (Hemiptera, Heteroptera, Reduviidae). Infect. Genet. Evol..

[141] Villegas J., Feliciangeli M.D., Dujardin J.P. (2002). Wing shape divergence between *Rhodnius prolixus* from Cojedes (Venezuela) and *R. robustus* from Mérida (Venezuela). Infect. Genet. Evol..

[142] Gurgel-Goncalves R., Abad-Franch F., Ferreira J., Santana D., Cuba C. (2008). Is *Rhodnius prolixus* (Triatominae) invading houses in central Brazil ?. Acta Trop..

[143] Márquez E.J., Jaramillo-O N., Gómez-Palacio A., Dujardin J.P. (2011). Morphometric and molecular differentiation of a *Rhodnius robustus*-like form from *R. robustus* Larousse, 1927 and *R. prolixus* Stal, 1859 (Hemiptera, Reduviidae). Acta Trop..

[144] Noireau F., dos Santos S.M., Gumiel M., Dujardin J.P., dos Santos Soares M., Carcavallo R.U., Galvão C., Jurberg J. (2002). Phylogenetic relationships within the oliveirai complex (Hemiptera: Reduviidae: Triatominae). Infect. Genet. Evol..

[145] Gonzalez-Britez N.E., Carrasco H.J., Martínez Purroy C.E., Feliciangeli D., Maldonado M., López E., Segovia M.J., Rojas de Arias A. (2014). Genetic and morphometric variability of *Triatoma sordida* (Hemiptera: Reduviidae) from the eastern and western regions of Paraguay. Front. Public Health.

[146] Klingenberg C.P., Gidaszewski N.A. (2010). Testing and Quantifying Phylogenetic Signals and Homoplasy in Morphometric Data. Syst. Biol..

[147] Varón-González C., Whelan S., Klingenberg C.P. (2020). Estimating Phylogenies from Shape and Similar Multidimensional Data: Why It Is Not Reliable. Syst. Biol..

[148] Schofield C.J., Galvão C. (2009). Classificatión, evolutión, and species groups within the Triatominae. Acta Trop..

[149] Hwang W.S., Weirauch C. (2012). Evolutionary History of Assassin Bugs (Insecta: Hemiptera: Reduviidae): Insights from Divergence Dating and Ancestral State Reconstruction. PLoS ONE.

[150] Patterson J.S., Schofield C.J., Dujardin J.P., Miles M.A. (2001). Population morphometric analysis of the tropicopolitan bug *Triatoma rubrofasciata* and relationships with Old World species of *Triatoma*: Evidence of New World ancestry. Med. Vet. Entomol..

[151] Dujardin J.P., Chavez T., Moreno J.M., Machane M., Noireau F., Schofield C.J. (1999). Comparison of isoenzyme electrophoresis and morphometric analysis for phylogenetic reconstruction of the Rhodniini (Hemiptera: Reduviidae: Triatominae). J. Med. Entomol..

[152] Almeida C.E., Marcet P.L., Gumiel M., Takiya D.M., Cardozo-de Almeida M., Pacheco R.S., Lopes C.M., Dotson E.M., Costa J. (2009). Phylogenetic and phenotypic relationships among *Triatoma carcavalloi* (Hemiptera: Reduviidae: Triatominae) and related species collected in domiciles in Rio Grande do Sul State, Brazil. J. Vector Ecol..

[153] Panzera F., Dujardin J.P., Nicolini P., Caraccio M.N., Rose V., Tellez T., Bermudez H., Bargues M.D., Mas-Coma S., O’Connor J.E. (2004). Genomic changes of Chagas disease vector, South America. Emerg. Infect. Dis..

[154] Dujardin J.P., Muñoz M., Chavez T., Ponce C., Moreno J., Schofield C.J. (1998). The origin of *Rhodnius prolixus* in Central America. Med. Vet. Entomol..

[155] Monroy C., Bustamante D.M., Rodas A., Rosales R., Mejia M., Tabaru Y. (2003). Geographic distribution and morphometric differentiation of *Triatoma nitida* Usinger 1939 (Hemiptera: Reduviidae: Triatominae) in Guatemala. Mem. Inst. Oswaldo Cruz.

[156] Piccinali R.V., Gaspe M.S., Nattero J., Gürtler R.E. (2023). Population structure and migration in *Triatoma infestans* (Hemiptera: Reduviidae) from the Argentine Chaco: An integration of genetic and morphometric data. Acta Trop..

[157] Gómez-Palacio A., Jaramillo-O N., Caro-Riaño H., Diaz S., Monteiro F., Pérez R., Panzera F., Triana O. (2012). Morphometric and molecular evidence of intraspecific biogeographical differentiation of *Rhodnius pallescens* (Hemiptera: Reduviidae: Rhodniini) from Colombia and Panama. Infect. Genet. Evol..

[158] Vendrami D.P., Obara M.T., Gurgel-Gonçalves R., Ceretti-Junior W., Marrelli M.T. (2017). Wing geometry of *Triatoma sordida* (Hemiptera: Reduviidae) populations from Brazil. Infect. Genet. Evol..

[159] Nattero J., Pita S., Calleros L., Crocco L., Panzera Y., Rodríguez C., Panzera F. (2016). Morphological and Genetic Differentiation within the Southernmost Vector of Chagas Disease: *Triatoma patagonica* (Hemiptera– Reduviidae). PLoS ONE.

[160] Noireau F., Flores R., Gutierrez T., Dujardin J.P. (1997). Detection of silvatic dark morphs of *Triatoma infestans* in the Bolivian Chaco. Memórias Inst. Oswaldo Cruz.

[161] Dujardin J.P., Bermudez H., Casini C., Schofield C.J., Tibayrenc M. (1997). Metric differences between silvatic and domestic *Triatoma infestans* (Hemiptera, Reduviidae) in Bolivia. J. Med. Entomol..

[162] Dujardin J.P., Beard C.B., Ryckman R. (2007). The relevance of wing geometry in entomological surveillance of Triatominae, vectors of Chagas disease. Infect. Genet. Evol..

[163] Gaspe M.S., Provecho Y.M., Piccinali R.V., Gürtler R.E. (2015). Where do these bugs come from? Phenotypic structure of *Triatoma infestans* populations after control interventions in the Argentine Chaco. Mem. Inst. Oswaldo Cruz.

[164] Jaramillo N., Castillo D., Wolff M. (2002). Geometric morphometric differences between *Panstrongylus geniculatus* from field and laboratory. Mem. Inst. Oswaldo Cruz.

[165] Gurgel-Gonçalves R., Maeda M., Ferreira J., Rosa A., Cuba C. (2011). Morphometric changes of *Rhodnius neglectus* (Hemiptera: Reduviidae) in the transition from sylvatic to laboratory conditions. Zoologia.

[166] Paschoaletto L., Santos-Mallet J., Dale C., Neiva V., Macedo Lopes C., Costa J. (2020). A 72 years of temporal analysis through geometric morphometrics detects phenotypic variationin populations of *Triatoma infestans* (Klug, 1834). Acta Biol. Par..

[167] Ribeiro G., Reis J., Vaccarezza F., Silva A.C.O., Lanza F.C., Miranda D.L.P., Gurgel-Gonçalves R., Reis M.G.D. (2023). Sometimes, the size matters: Wing geometric morphometrics as a tool to assess domiciliation by *Triatoma sordida* (Stäl 1859). Rev. Soc. Bras. Med. Trop..

[168] Vargas E., Espitia C., Patińo C., Pinto N., Aguilera G., Jaramillo C., Bargues M.D., Guhl F. (2006). Genetic structure of *Triatoma venosa* (Hemiptera: Reduviidae): Molecular and morphometric evidence. Mem. Inst. Oswaldo Cruz.

[169] Usinger R.L., Wygodzinsky P., Ryckman R.E. (1966). The Biosystematics of Triatominae. Annu. Rev. Entomol..

[170] Nattero J., Mougabure-Cueto G., Gürtler R.E. (2022). Sublethal effects of a pyrethroid insecticide on cuticle thickness, wing size and shape in the main vector *Triatoma infestans*. Med. Vet. Entomol..

[171] Gaspe M.S., Schachter-Broide J., Gurevitz J.M., Kitron U., Gürtler R.E., Dujardin J.P. (2012). Microgeographic Spatial Structuring of *Triatoma infestans* (Hemiptera: Reduviidae) Populations Using Wing Geometric Morphometry in the Argentine Chaco. J. Med. Entomol..

[172] Jirakanjanakit N., Leemingsawat S., Dujardin J.P. (2008). The geometry of the wing of *Aedes* (*Stegomyia*) *aegypti* in isofemale lines through successive generations. Infect. Genet. Evol..

[173] Kamimura E., Viana M.C., Lilioso M., Fontes F., Pires da Silva D., Valença-Barbosa C., Carbajal de la Fuente A., Folly-Ramos E., Solferin V., Thyssen P. (2020). Drivers of molecular and morphometric variation in *Triatoma brasiliensis (Hemiptera: Triatominae)*: The resolution of geometric morphometrics for populational structuring on a microgeographical scale. Parasites Vectors.

[174] Dumonteil E., Tripet F., Ramirez-Sierra M.J., Payet V., Lanzaro G., Menu F. (2007). Assessment of *Triatoma dimidiata* dispersal in the Yucatan peninsula of Mexico by morphometry and microsatellite markers. Am. J. Trop. Med. Hyg..

[175] Feliciangeli M.D., Sanchez-Martin M., Marrero R., Davies C., Dujardin J.P. (2007). Morphometric evidence for a possible role of *Rhodnius prolixus* from palm trees in house re-infestation in the State of Barinas (Venezuela). Acta Trop..

[176] Ceballos L.A., Piccinali R.V., Marcet P.L., Vazquez-Prokopec G.M., Cardinal M.V., Schachter-Broide J., Dujardin J.P., Dotson E.M., Kitron U., Gürtler R.E. (2011). Hidden Sylvatic Foci of the Main Vector of Chagas Disease *Triatoma infestans*: Threats to the Vector Elimination Campaign?. PLoS Negl. Trop. Dis..

[177] Hernández M.L., Dujardin J.P., Gorla D.E., Catalá S.S. (2013). Potential sources of *Triatoma infestans* reinfesting peridomiciles identified by morphological characterization in Los Llanos, La Rioja, Argentina. Mem. Inst. Oswaldo Cruz.

[178] Gaspe M.S., Gurevitz J.M., Gürtler R.E., Dujardin J.P. (2013). Origins of house reinfestation with *Triatoma infestans* after insecticide spraying in the Argentine Chaco using wing geometric morphometry. Infect. Genet. Evol..

[179] Nattero J., Cecere M.C., Gürtler R.E. (2017). Temporal variations of fluctuating asymmetry in wing size and shape of *Triatoma infestans* populations from northwest Argentina. Infect. Genet. Evol..

[180] Paschoaletto L., Dale C., Neiva V., Carbajal de la Fuente A., Oliveira J., Benítez H., Costa J. (2022). Morphological Stasis in Time? A *Triatoma brasiliensis brasiliensis* Study Using Geometric Morphometrics in the Long Run. Animals.

[181] Schofield C.J., Service M.W. (1988). Biosystematics of the Triatominae. Biosystematic of Haematophagous Insects.

[182] Vilaseca C., Méndez M.A., Pinto C.F., Benítez H.A. (2020). Assessment of Shape Variation Patterns in *Triatoma infestans* (Klug 1834) (Hemiptera: Reduviidae: Triatominae): A First Report in Populations from Bolivia. Insects.

